# Epidemiology of brain abscess in Taiwan: A 14-year population-based cohort study

**DOI:** 10.1371/journal.pone.0176705

**Published:** 2017-05-09

**Authors:** Cheung-Ter Ong, Ching-Fang Tsai, Yi-Sin Wong, Solomon Chih-Cheng Chen

**Affiliations:** 1 Department of Neurology, Ditmanson Medical Foundation Chiayi Christian Hospital, Chiayi City, Taiwan; 2 Department of Nursing, Chung Jen Junior College of Nursing, Health Science and Management, Chiayi City, Taiwan; 3 Department of Medical Research, Ditmanson Medical Foundation Chiayi Christian Hospital, Chiayi City, Taiwan; 4 Department of Family Medicine, Ditmanson Medical Foundation Chiayi Christian Hospital, Chiayi City, Taiwan; 5 Department of Pediatrics, School of Medicine, Taipei Medical University, Taipei, Taiwan; Aga Khan University Hospital Nairobi, KENYA

## Abstract

Brain abscess (BA) is a severe neurological emergency, which remains a challenge for physicians despite medical advancements. The purpose of this study is to describe the epidemiology of BA in Taiwan and to investigate potential factors affecting the survival of patients with BA. By using the Taiwan National Health Insurance Research Database, we identified hospitalized patients with a discharge diagnosis of pyogenic BA (324.X) between 2000 and 2013. The incidence and in-hospital mortality of BA were calculated based on both age and sex. A total of 6027 BA cases were identified. The overall incidence of BA was 1.88 (95% CI: 1.83–1.93) per 100,000 person-years and increased with age, from 0.58 per 100,000 person-years in individuals aged 0–14 years to 4.67 per 100,000 person-years in those over 60 years of age. The male-to-female incidence ratio was 2.37 (95% CI: 2.24–2.50), with a mountain-shaped distribution across ages peaking at 40–44 years. The in-hospital mortality also increased with age, from 4.22% (95% CI: 2.54–6.97) at 0–14 years to 17.34% (95% CI: 15.79–19.02) in individuals over 60 years of age, without a gender difference (11.9% for males, 12.5% for females). Age, stroke, septicemia, pneumonia, meningitis, and hepatitis were associated with increased risk of in-hospital mortality. There was a male predominance for BA, and both the incidence and in-hospital mortality rates increased with age. Infection-related disease such as septicemia, pneumonia and meningitis were important factors associated with in-hospital mortality. In addition to the original treatment of BA, we suggest paying close attention to potential infections to improve the outcome of BA patients.

## Introduction

Brain abscess (BA) is a rare but life-threatening disease. The main strategies for BA treatment include surgical intervention and antibiotic therapy. Although previous studies examined various characteristics of BA, such as age, gender, location, symptoms, pathogens, and outcomes [1–6], few studies have assessed the incidence of BA. In addition, these studies included only a small number of cases [7, 8]. One recent study, which was conducted in Olmsted County, Minnesota, United States, reported the incidence of BA as 2.7 per 100,000 from 1935–1944 and 0.9 per 100,000 from 1965–1981[7].

Recently, newly diagnostic procedures, such as brain imaging techniques (i.e., magnetic resonance imaging [MRI] and computed tomography [CT]) and stereotactic biopsy, as well as administration of new antibiotics have considerably changed the management and outcome of patients with BA [2, 4, 8, 9]. The mortality of BA has declined from 40% in 1960 to 10%–20% during the past decade [1–4, 9, 10]. Unfortunately, many survivors continue to suffer from neurological deficits [1]. Moreover, most studies on BA outcomes focused on advances in new diagnostic procedures and neurosurgery, causative organisms, location, and clinical symptoms [7, 9–13]. No study investigated the impact of comorbidities on BA outcomes.

We are interested in whether improvements in the diagnosis and management of BA have changed the mortality rates of BA. This study aims to describe the trend in BA incidence over a 14-year period and to investigate the potential impact of predisposing factors and comorbidities on BA mortality.

## Materials and methods

### Data source and ethical approval

This investigation was a population-based cohort study using data obtained from the Taiwan National Health Insurance Research Database (NHIRD). The NHIRD is a claim dataset that has been used extensively for many studies [10–12]. Established in 1995, the National Health Insurance system in Taiwan is compulsory for all Taiwanese residents except for criminals and military personnel and covers over 99% of the total population of approximately 23 million people. The NHIRD contains registration files and original claims data for reimbursement, including records of demographic data; dates of clinical visits; diagnostic codes; details of prescriptions, examinations, and procedures; and medical expenditures. In each admission record, up to five discharge diagnoses are coded according to the International Classification of Disease, Ninth Revision, Clinical Modification (ICD-9-CM). This study was approved by the Institutional Review Board of the Ditmanson Medical Foundation Chiayi Christian Hospital, Taiwan (CYCH-IRB: 105003).

### Study subjects and definitions

A BA was defined as a collection of purulent material within the cranial cavity that was confirmed by imaging (CT or MRI), biopsy, or surgery [9]. All patients who were hospitalized due to BA from January 1, 2000, to December 31, 2013, were included in the study. An ICD-9-CM code of 324.X, denoted as one of the first three diagnoses, was used to identify patients with BA. In patients with neurosurgical intervention, all materials from BA were cultured for aerobic and anaerobic bacteria, mycobacteria and fungi. In the patient without neurosurgical intervention, blood culture was performed for aerobic and anaerobic bacteria, mycobacteria and fungi. The age and sex of patients, calendar year of hospitalization, and in-hospital mortality related to BA were studied. The age at first diagnosis was categorized into five groups: 0–14, 15–29, 30–44, 45–59, and over 60 years of age. Comorbidities and predisposing factors were ascertained from the ICD-9-CM codes of the first five discharge diagnoses and the most common predisposing factors and comorbidities were included in further analyses.

### Statistical analysis

A Chi-squared test was performed to compare the frequency of different variables between survival and deceased cases, whereas a Student’s t test was used to compare average values between the groups. A Wilcoxon rank-sum test was used to compare the median days of hospitalization. Multivariate logistic regression analysis was performed to estimate the odds ratio (OR) and 95% confidence intervals (CI) of in-hospital fatality of patients with BA. A *p-*value of less than .05 was considered to be statistically significant. Data management and analyses were performed using SAS/STAT^®^ software, version 9.3 for Windows (SAS Institute Inc., Cary, NC, USA).

## Results

### Incidence and sex ratio

**Fig 1:**
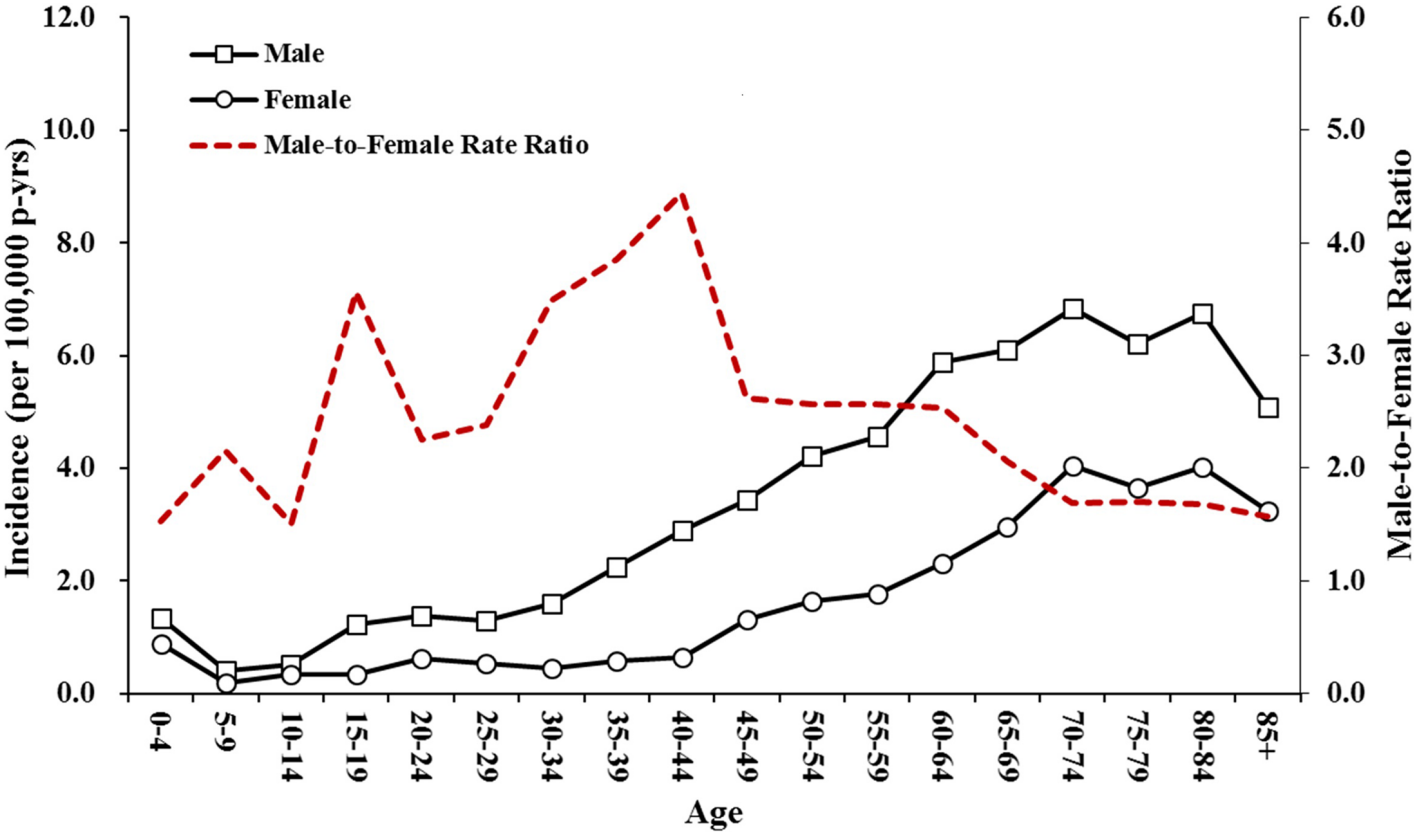
The incidence and male-to-female rate ratio of brain abscess by age in Taiwan from 2000 to 2013.

During the period between 2000 and 2013, there were 6027 patients hospitalized with BA (4265 males, 1762 females) in Taiwan. The overall incidence rate was 1.88 (95% CI: 1.83–1.93) per 100,000 person-years (Table 1). The incidence was lowest in the 0–14 year age group and increased with age (Table 1 and Fig 1). There was a male predominance (male-to-female rate ratio = 2.37; Table 1). The male-to-female incidence ratio changed with age and showed a mountain-shaped distribution with a peak at 40–44 years of age (Fig 1).

**Table 1:** Incidence rate (per 100,000 person-years) of hospitalized brain abscess in Taiwan, 2000–2013.

Parameter	No. of case	%	Incidence Rate (95% CI)	Relative risk (95% CI)
All patients	6027	100.00	1.88 (1.83–1.93)	-
Sex				
Female	1762	29.24	1.11 (1.10–1.16)	Reference
Male	4265	70.76	2.63 (2.55–2.71)	2.37 (2.24–2.50)
Age, y				
0–14	331	5.49	0.58 (0.52–0.65)	Reference
15–29	677	11.23	0.91 (0.85–0.98)	1.57 (1.38–1.79)
30–44	1115	18.80	1.41 (1.33–1.49)	2.42 (2.14–2.73)
45–59	1800	29.87	2.77 (2.64–2.90)	4.75 (4.23–5.34)
≥60	2104	34.91	4.67 (4.47–4.87)	8.01 (7.13–8.99)
Year				
2000–2002	1158	19.21	1.72 (1.63–1.83)	Reference
2003–2005	1346	22.33	1.98 (1.87–2.09)	1.15 (1.06–1.24)
2006–2008	1349	22.38	1.96 (1.86–2.07)	1.14 (1.05–1.23)
2009–2011	1349	22.38	1.94 (1.84–2.05)	1.13 (1.04–1.22)
2012–2013	825	16.69	1.77 (1.65–1.89)	1.03(0.94–1.12)

### Age distribution of in-hospital mortality

**Fig 2:**
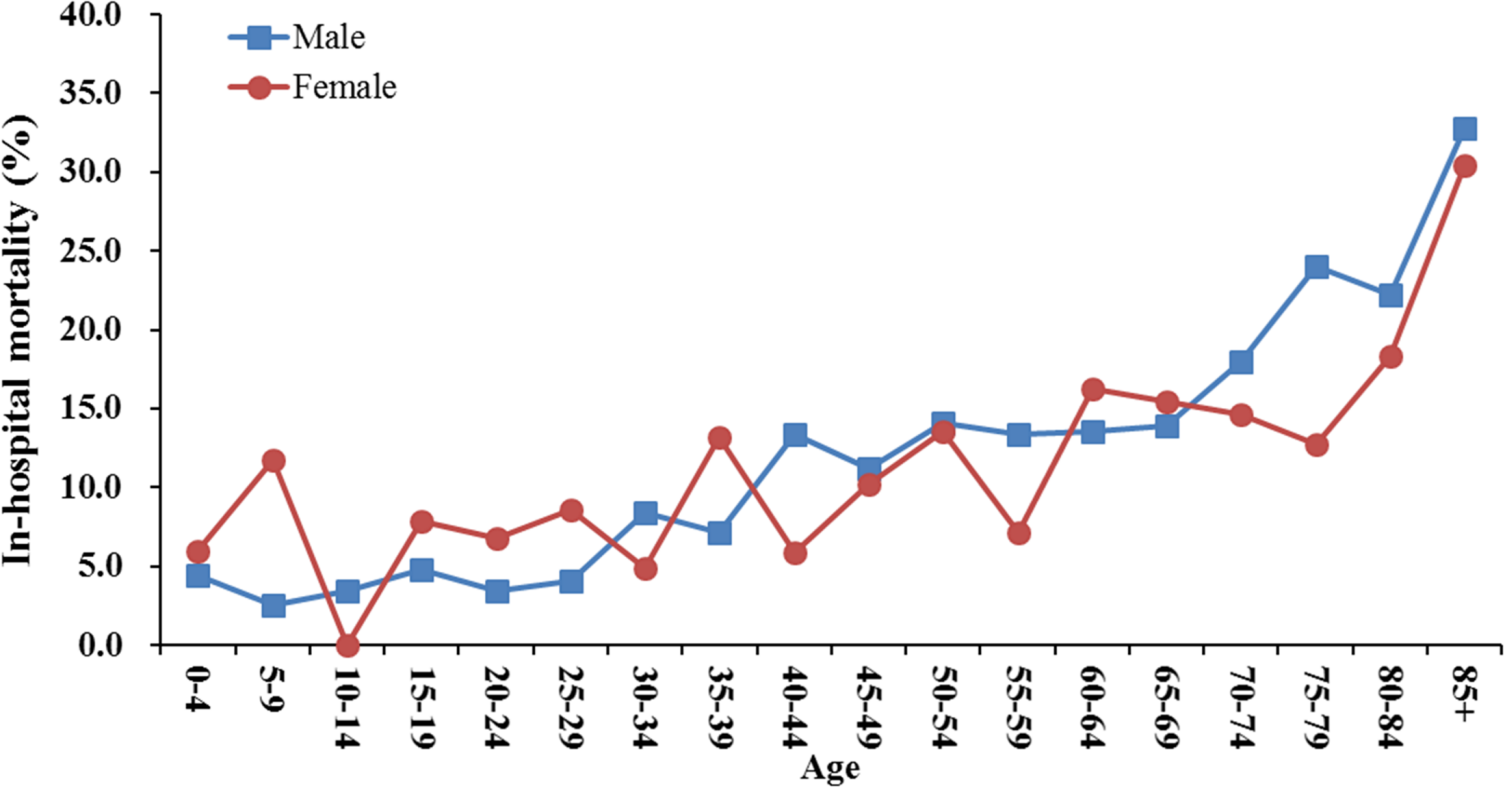
In-hospital mortality of brain abscess by age in Taiwan from 2000 to 2013.

Among all cases, there were 741 deaths (531 males, 210 females), with an overall in-hospital mortality rate of 12.29% (95% CI: 11.49–13.15). The mortality rate also increased with age, from 4.22% (95% CI: 2.54–6.97) in patients 0–14 years of age to 17.34% (95% CI: 15.79–19.02) in patients older than 60 years of age (Fig 2). The mortality rate was similar between the two sexes (11.92% for males and 12.45% for females; Table 2). In addition, it revealed a declining trend from 13.0% to 10.2% over the study period, though without statistical significance (Table 2). The overall length-of-stay (LOS) in the hospital was 45.7 ± 45.5 days, with longer stays in the survival group than in the mortality group (47.3 ± 46.2 vs. 34.5 ± 38.5 days, respectively, *P* < .001; Table 2).

**Table 2:** Demographic characteristics comparison between survival and death cases.

Variables -No.(%)	Total	Survival	In-hospital death	P value
All patients	6027	5286 (87.7%)	741 (12.3%)	
Sex				
Male	4265	3734 (87.5%)	531 (12.5%)	0.8360^a^
Female	1762	1552 (88.1%)	210 (11.9%)	
Age, years				
mean±SD	50.6±20.6	49.5±20.3	58.5±18.1	<0.0001^b^
0–14	331	317 (95.7%)	14 (4.3%)	<0.0001^a^
15–29	677	643 (95.0%)	34 (5.0%)	
30–44	1115	1007 (90.3%)	108 (9.7%)	
45–59	1800	1580 (87.8%)	220 (12.2%)	
≥60	2104	1739 (80.5%)	365 (19.5%)	
Year				
2000–2002	1158	1007 (87.0%)	151 (13.0%)	0.2681^a^
2003–2005	1346	1168 (86.8%)	178 (13.2%)	
2006–2008	1349	1183 (87.7%)	166 (12.3%)	
2009–2011	1349	1187 (88.0%)	162 (12.0%)	
2012–2013	825	741 (89.8%)	84(10.2%)	
LOS, days				
mean±SD	45.7±45.5	47.3±46.2	34.5±38.5	<0.0001^b^
median (Q1-Q3)	37 (18–59)	39 (20–60)	23 (10–46)	<0.0001^c^
Predisposing factors				
Septicemia	593	349(58.9%)	244(41.1%)	<0.0001^a^
Meningitis	474	386(81.4%)	88(18.6%)	<0.0001^a^
Pneumonia	415	305(73.5%)	112(26.5%)	<0.0001^a^
Systemic infection	413	387(93.7%)	26(6.3%)	0.0001^a^
Comorbidity				
Pulmonary disease	824	537 (65.2%)	287 (34.8%)	<0.0001^a^
Diabetes mellitus	866	735 (84.9%)	131 (15.1%)	0.0061^a^
Hypertension	788	732(92.9%)	56(7.1%)	<0.0001^a^
Hepatitis	420	330(78.6%)	90(21.4%)	<0.0001^a^
Other factors				
Head injury	721	688 (95.4%)	33 (4.6%)	<0.0001^a^
Stroke	623	494 (79.3%)	129 (20.7%)	<0.0001^a^
Brain tumor	255	225(88.2%)	30(11.8%)	0.4347^a^

SD: standard deviation; IQR: inter-quartile range.

^a^Chi-Square.

^b^Student’s *t* test.

^c^Wilcoxon rank-sum test

### Predisposing factors and comorbidities

Among all 6027 cases, the most common predisposing factors were septicemia in 593, meningitis in 474, pneumonia in 415 and systemic infection in 387 patients. The most common comorbidities were pulmonary disease in 824, diabetes mellitus in 866, hypertension in 788 and hepatitis in 420 patients (Table 2). Further analysis by multivariate logistic regression revealed that septicemia, pneumonia and meningitis all increase the risk of in-hospital mortality. The comorbidities, pulmonary disease and hepatitis, were associated with an increased risk of in-hospital mortality, whereas hypertension and head injury were associated with a decreased risk of in-hospital mortality (Table 3). Traumatic brain injury related BA was 12.5% (531/4265) in men and 10.8% (190/1762) in women, were not statistically different (*p* = .07). Further analysis of the data stratified by sex showed septicemia, pneumonia, and pulmonary disease increase the risk of in-hospital mortality in both men and women. In contrast meningitis and stroke increase the risk of in-hospital mortality in men but not in women, whereas both head injury and hypertension decrease the risk of in-hospital mortality in men but not in women.

**Table 3:** Multivariate logistic regression analysis of in-hospital fatality of brain abscess cases.

Parameter	Case fatality (95% CI), %	Univariate		Multivariate	
		OR	95% CI	OR	95% CI
All patients	12.29 (11.49–13.15)	-	-	-	-
Sex					
Female	12.45 (11.49–13.48)	Reference	-	Reference	
Male	11.92 (10.49–13.52)	1.05	0.89–1.25	1.10	0.91–1.34
Age, y					
0–14	4.22 (2.54–6.97)	Reference	-	Reference	
15–29	5.02 (3.62–6.94)	1.20	0.63–2.26	1.98	1.01–3.89
30–44	9.69 (8.09–11.56)	2.43	1.37–4.30	3.11	1.69–5.74
45–59	12.22 (10.79–13.82)	3.15	1.81–5.48	3.84	2.12–659
≥60	17.34 (15.79–19.02)	4.75	2.75–8.21	5.68	3.15–10.25
Year					
2000–2002	13.04 (11.22–15.10)	Reference	-	Reference	
2003–2005	13.22 (11.52–15.14)	1.02	0.81–1.28	0.95	0.73–1.22
2006–2008	12.31 (10.66–14.17)	0.94	0.74–1.19	0.81	0.63–1.06
2009–2011	12.00 (10.38–13.85)	0.91	0.72–1.15	0.78	0.60–1.02
2012–2013	10.18(8.30–12.43)	0.76	0.57–1.00	0.65	0.47–0.88
Predisposing factors					
Septicemia	41.14 (37.26–45.15)	6.95	5.76–8.38	5.61	4.56–6.90
Meningitis	18.57 (15.32–22.31)	1.71	1.34–2.19	1.56	1.18–2.06
Pneumonia	26.51 (22.49–30.95)	2.85	2.26–3.60	1.73	1.33–2.26
Systemic infection	6.30 (4.33–9.06)	0.46	0.31–0.69	0.70	0.46–1.07
Comorbidity					
Pulmonary disease	34.83 (31.65–38.15)	5.59	4.70–6.64	3.78	3.12–4.58
Hypertension	7.11 (5.51–9.12)	0.51	0.38–0.68	0.60	0.44–0.81
Diabetes mellitus	15.13 (12.90–17.67)	1.33	1.08–1.63	1.22	0.97–1.53
Hepatitis	21.43 (17.77–25.61)	2.08	1.62–2.66	2.11	1.60–2.79
Other factors					
Stroke	20.71 (17.71–24.06)	2.05	1.66–2.53	1.66	1.31–2.10
Head injury	4.58 (3.28–6.36)	0.31	0.22–0.45	0.50	0.34–0.72
Brain tumor	11.76 (8.37–16.30)	0.95	0.64–1.40	1.27	0.84–1.94

Multivariable logistic regression analysis

## Discussion

This nationwide, population-based study on BA revealed a steady annual incidence (1.72–1.98 per 100,000 person-years) and a slight decline in mortality rates from 13.0% to 10.2% throughout the study period. The incidence of BA had a male predominance (male-to-female rate ratio of 2.37) and increased with age. The in-hospital fatality also increased with age but without a gender difference. Lastly, the multivariate regression analysis found that concomitant infection-related predisposing factors, such as septicemia, meningitis, and pneumonia, might increase the risk of in-hospital mortality in patients with BA.

The incidence rate in this study was higher than that reported previously [7, 8]. The incidence of BA was 3–5 per million population-years in a study conducted in Northern Ireland [8], which identified BA cases based on pathology and excluded patients with abscesses smaller than 15 mm in diameter. A more recent investigation conducted in Olmsted County in Minnesota showed a decline in the incidence of BA from 2.7 per 100,000 persons during 1935–1944 to 0.9 per 100,000 persons during 1965–1981 [7]. However, these studies were performed over 20 years ago in Western countries [7, 8]. The difference in the incidence between previous studies and the present study could be due to different inclusion criteria, ethics, and advances in diagnostic technology introduced during recent decades [13–16].

The male predominance in BA noted in this study was consistent with previous studies [1–4, 7, 8]. Moreover, the dynamic change in the male-to-female rate ratio of BA cases, which exhibited a mountain-shaped distribution and a peak at 40–45 years of age, was similar to our previous study on the incidence of status epilepticus, which also showed a mountain-shaped distribution of the male-to-female rate ratio [12]. It is noteworthy that this age period corresponds to the female premenopausal period since the protective effects of estrogen in neurons were proposed previously [17]. However, the role of reproductive hormones in the development of BA needs further investigation.

There are several predisposing factors of BA such as being immunocompromised, having existing medical conditions, and distant infection [1, 9, 18]. Sharma *et al*. reported that an adjacent focus of infection (i.e., sinusitis, otogenic, odontogenic, and post-meningitis) was found in 42.5% of patients with BA, followed by neurosurgical procedures and distant infections [4]. Nathoo *et al*. reported that otorhinogenic infections (38.5%) and trauma (32.8%) were the two most common causes of BA in South Africa [16]. Otorhinogenic infection, which appears to be the most important etiology of BA, is often observed in the first decade of life [2, 4, 16, 19]. However, its contribution to the incidence of BA was only 3.1% (185/6,027) in the present study, which might explain the low number of BA cases found in the young age group in Taiwan (Fig 1). The age-specific incidence of BA in this study was similar to a report by Tsou *et al*. [3], but was different from previous studies conducted in Olmsted County and South Africa [7, 16]. In the Olmsted County study, the highest incidence of BA was in the 5–9 year age group [7]. Similarly, in the study from South Africa, approximately 70% of patients were in the first three decades of life, and 43% were younger than 18 years of age [16]. Taken together, we propose that the low number of BA cases in the young age group may be associated with fewer severe otorhinogenic infections in Taiwan. Because most patients can access health care conveniently that would reduce the spread of pathogens from otorhinogenic infections to the brain.

The in-hospital mortality rate of BA in our analysis (12.3%) was similar to the recently reported rates of 9%–15% [2, 14, 16]. However, it was much lower than the 38–53% of earlier reports [6–8, 20]. Most previous studies were based on a single or small number of institutes, unlike the present study that use a national representative sample. One rational finding was the fatality rate increased with age. However, despite the higher incidence of BA in males, the case-fatality rate was similar in both genders. This finding is in agreement with previous studies that showed no gender difference in BA mortality [7, 16, 21]. In addition, we found a significantly longer LOS in the hospital for those who survived compared with those who died. This result might be because survivors usually suffer from neurological sequelae that need subsequent treatment in the hospital, whereas fatal cases likely followed a faster clinical course occurring within a certain time period.

Preexisting comorbidities and predisposing factors can greatly affect the survival of patients with BA [7,19, 21,]. Our results, consistent with previous studies, found that infection-related factors, such as meningitis [18], pneumonia and septicemia [21] were associated with increased mortality. Consistent with the finding of previous studies that pulmonary disease and hepatitis can increase the risk of in-hospital mortality in trauma patients [22, 23]; our study found pulmonary disease and hepatitis increase the risk of in-hospital mortality in BA patients. In contrast, non-infection-related comorbidities did not affect BA mortality. For example, head injury patients had an even lower fatality rate [16], as head injuries occur more frequently in younger patients, who are usually healthier prior to the BA episode, and are mostly due to traffic accidents, [24]. Hypertension may change the upper and lower blood pressure limit of brain autoregulation. A previous study showed that during hypoxia, the cerebral blood flow is higher in hypertensive patients than in normotensive patients [25]. Lower fatality rate in BA patients with hypertension suspected related to hypertensive patients have more cerebral blood flow than normotensive patients. However, this needs further investigation.

There are several limitations of this study. First, case determination was based on the claims dataset, which might raise the question of diagnostic accuracy. Taiwan has a comprehensive healthcare coverage with the national health insurance system. All patients with suspected BA are usually referred to hospitals with available neurologists, where the diagnosis can be confirmed by brain CT or MRI and pus or blood culture. Therefore, the coding of BA is highly reliable. Second, various factors affect mortality of BA, such as consciousness at the time of admission, nature of infection, and location and multiplicity of abscesses [2, 8, 16, 26]. However, the NHIRD as a claims dataset was not designed for academic research, and clinical information on symptoms, number, location, and size of lesions, and mode of neurosurgical intervention was not available in the study dataset. In addition, data on the pathogenic etiology of BA, such as the species of bacteria, fungi, parasite, culture rate, and functional outcome, was not available and remains beyond the scope of the current study.

## Conclusions

This nationwide population cohort study revealed the epidemiological information about BA in Taiwan. Both the incidence and mortality rates both increased with age. Although incidence had a male predominance, mortality showed no sex difference. Infection-related diseases such as septicemia, pneumonia, and meningitis, significantly increased the risk of in-hospital mortality. In contrast, non-infection-related comorbidities, such as diabetes mellitus and a brain tumor, did not affect mortality. Therefore, we suggest paying attention to potential infections to improve the outcome of BA patients.

## Supporting information

S1 Table Calendar year trend of Incidence rate (per 100,000 person-years) stratified by age and sex. (DOC)

## Abbreviations

CI: confidence interval
ICD-9-CM: International Classification of Diseases, 9^th^ Revision, Clinical Modification
NHIRD: National Health Insurance Research Database
OR: odds ratio
