# Supplementary material for: Epidemiology of brain abscess in Taiwan: A 14-year population-based cohort study
Source: PLoS One. 2017 May 9;12(5):e0176705. doi: 10.1371/journal.pone.0176705 (PMC5423610; doi:10.1371/journal.pone.0176705)
Supplement: S1 Table — (DOC) [file pone.0176705.s001.doc]

S1 Table: Calendar year trend of Incidence rate (per 100,000 person-years) stratified by age and sex

| Incidence | 2000-2002 | 2003-2005 | 2006-2008 | 2009-2011 | 2012-2013 | P value for trend |
| --- | --- | --- | --- | --- | --- | --- |
| Sex |  |  |  |  |  |  |
| Female | 1.06 | 1.14 | 1.16 | 1.13 | 1.05 | 0.9450 |
| Male | 2.36 | 2.78 | 2.74 | 2.75 | 2.48 | 0.2483 |
| Age, y |  |  |  |  |  |  |
| 0-14 | 0.62 | 0.66 | 0.61 | 0.49 | 0.46 | 0.0535 |
| 15-29 | 1.00 | 1.00 | 0.98 | 0.83 | 0.64 | 0.0019 |
| 30-44 | 1.28 | 1.50 | 1.50 | 1.45 | 1.27 | 0.9638 |
| 45-59 | 2.77 | 2.90 | 2.79 | 2.80 | 2.50 | 0.2182 |
| ≥60 | 4.54 | 5.32 | 4.87 | 4.54 | 3.99 | 0.0119 |
